# Nanotechnology-Based Strategies to Overcome Current Barriers in Gene Delivery

**DOI:** 10.3390/ijms22168537

**Published:** 2021-08-09

**Authors:** Sofía Mirón-Barroso, Elena B. Domènech, Sonia Trigueros

**Affiliations:** 1 Institute of Molecular Biology of Barcelona, IBMB-CSIC, 08028 Barcelona, Spain; smbcri@ibmb.csic.es; 2 Department of Genetics Microbiology and Statistics, University of Barcelona, 08007 Barcelona, Spain; elenabdomenech@gmail.com; 3 Department of Zoology, University of Oxford, Oxford OX1 3PS, UK

**Keywords:** nanotechnology, nanomaterials, nanomedicine, delivery, gene therapy, genetic material, gene therapeutics, biological barriers, cancer

## Abstract

Nanomaterials are currently being developed for the specific cell/tissue/organ delivery of genetic material. Nanomaterials are considered as non-viral vectors for gene therapy use. However, there are several requirements for developing a device small enough to become an efficient gene-delivery tool. Considering that the non-viral vectors tested so far show very low efficiency of gene delivery, there is a need to develop nanotechnology-based strategies to overcome current barriers in gene delivery. Selected nanostructures can incorporate several genetic materials, such as plasmid DNA, mRNA, and siRNA. In the field of nanotechnologies, there are still some limitations yet to be resolved for their use as gene delivery systems, such as potential toxicity and low transfection efficiency. Undeniably, novel properties at the nanoscale are essential to overcome these limitations. In this paper, we will explore the latest advances in nanotechnology in the gene delivery field.

## 1. Introduction

Numerous diseases find their roots at the genetic level. The human genome project and the latest advances in molecular genetics and high throughput techniques allowed us to understand the genetic basis of many pathologies and, thus, to identify new therapeutic targets [1,2]. Therefore, new strategies are being developed for “undruggable” diseases [3]. In the past few decades, gene therapy emerged as a potential treatment for a wide range of diseases, including cancer, cardiovascular and neurological diseases [4]. However, due to the diseases and genetic defects’ heterogeneity, different molecular approaches have been developed to achieve the therapeutic goal.

## 2. Gene Therapy

**Figure 1:**
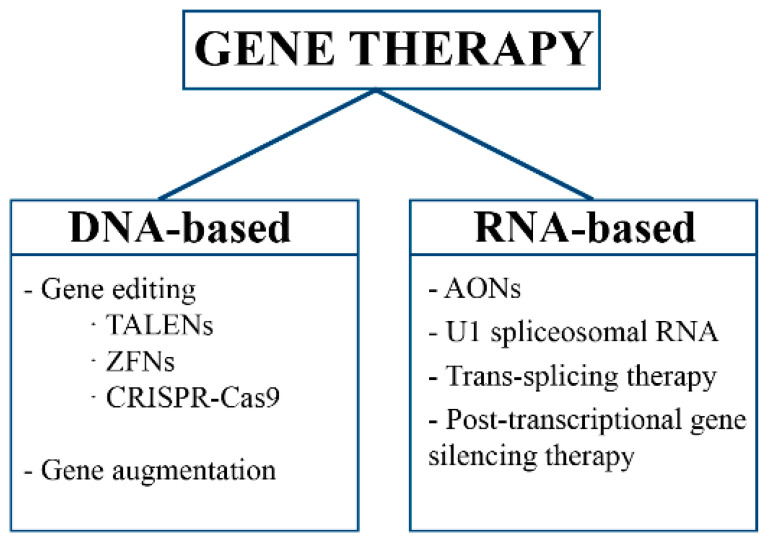
Classification of gene therapy approaches based on nucleic acid type.

Gene therapy is an experimental technique that modifies genes or gene expression to treat or prevent diseases. It can be used to restore cell function in monogenic disorders or to endow cells with new capabilities. Gene therapy works by editing, replacing, or altering gene expression instead of using drugs (Figure 1).

### 2.1. Gene Editing

Currently, the three main approaches to edit the genome are: zinc finger nucleases (ZFNs), transcription activator-like effector nucleases (TALENs), both of which correct the mutations ex vivo and clustered regularly interspaced short palindromic repeats (CRISPR)—CRISPR associated (Cas9) system (CRISPR/Cas9) which can correct mutations in vitro and in vivo [5,6]. These technologies introduce double-strand breaks in DNA-specific sites using enzymes that recognize a specific region of the genome. Gene editing occurs when cellular repair mechanisms correct the double-strand break by non-homologous end joining (NHEJ) which can introduce insertions or deletions or by homologous-directed repair (HDR) that needs a DNA template [6,7].

ZNFs encode a short monomer sequence thus, they are not limited by vector sequence capacity, but their limitations are related to the low number of sites they can effectively target, and the cytotoxicity produced when they generate an off-target. Mostly, the delivery systems used are viral; in particular, adeno-associated viruses, which contain a limited expression cassette of only 4.7 Kb capacity and also can produce a strong immune response [8,9].

While TALENs are less cytotoxic than ZNFs, the use of viral vectors has been difficult due to the bigger size of TALENs which make them challenging for a high efficiency cell delivery method. Non-viral delivery vectors are, therefore, the most suitable method of delivery for TALENs delivery due to the large cargo size capacity. As opposed to ZNFs, TALENs are not able to penetrate cell membranes when they are delivered without any vector [6,7].

Finally, CRISPR/Cas9 technology is the newest and more efficient system of gene editing. It can be performed by using DNA, RNA and/or protein. Choosing the delivery strategy depends on the application considering efficiency, toxicity and safety [10].

### 2.2. Gene Augmentation

This approach consists of replacing the mutant gene that is not functional by delivering the correct copy of the gene using a delivery vector. The therapeutic nucleic acid of interest can be DNA, mRNA, mRNA analogue or an oligonucleotide. The advantages of using RNA are the low risk of insertion in the host genome and that it does not need to be delivered in the nucleus. On the other hand, its low stability and the risk of immunogenicity are the main disadvantages. Currently, the most used approach is introducing the gene using plasmid DNA due to its high stability. Since plasmids become rapidly chromatinized once internalized, the gene enters the nucleus and remains episomal [11].

### 2.3. RNA Therapy

RNA therapeutics can either mimic or antagonize endogenous RNA functions. Several advantages of using RNA as a therapy consist of its ease of design, cost effectiveness, stability and easy combination with other drugs presenting also low immunogenicity [12]. The major RNA therapeutic methods are: (I) antisense oligonucleotides (AONs) which are small RNA or DNA chemically modified molecules that bind by complementary base pair to the pre-mRNA and their main functions are to exclude exons and pseudo-exons, include exons, degrade transcripts and block the translation [13]; (II) U1 spliceosomal RNA that utilizes a modified and an adapted U1 snRNA to the mutation favouring the correct splicing [5,12,14]; (III) trans-splicing therapy, that consists of introducing an exogen RNA containing a binding domain to the target mRNA and thus, activates the trans-splicing process [5,15]; and (IV) post-transcriptional gene silencing therapy which can be approached by iRNA to degrade the transcripts or by AONs RNAse H which are based on the hybrids RNA/DNA are degraded by RNAse H activity [12,16,17].

During the past few decades, gene therapy became an essential tool to treat multiple diseases and investigators strive to develop an efficient methodology. Although there are many clinical trials and some treatments are already approved, delivery systems for gene therapy are still a challenge. The therapeutic nucleic acids need to find the specific cell, enter the target cell, reach the nucleus without being degraded and finally be expressed or do its corrective function. Thus, the delivery system has to be directed to a specific cell, overcome physical cell barriers, avoid degradation and not cause toxicity to the body. These reasons raised the demand for a suitable delivery system that delivers gene therapeutics more efficiently, without toxicity and that are cell-targeted and cost-effective.

## 3. Why Does Gene Therapy Need a Carrier?

Gene therapeutics must cross many biological barriers in order to reach their site of action [18]. First of all, they need to reach their target organ/tissue/cells without being degraded. For instance, oligonucleotides have a very short half-life in physiological media due to the presence of endo- and exonucleases [19,20].

Depending on their target organ or tissue and their administration route, gene therapeutics must cross specific biological barriers. One of the most common administration routes is intravenous (IV). Once in circulation, gene therapeutics must cross the endothelium in order to reach their target. This barrier has different properties depending on the organ/tissue. Organs such as the spleen, liver or tumours have a fenestrated vasculature and thus, crossing the endothelium is easier when targeting these organs.

A vast number of studies aim to develop gene therapy strategies for the treatment of neurological diseases. In order for gene therapeutics to reach the brain, they must cross the blood–brain barrier (BBB) [21]. This biological barrier is a continuous endothelial membrane, present in the brain vasculature. Endothelial cells are attached to each other by tight junctions to avoid the crossing of substances such as toxins and pathological microorganisms [22].

Currently, 67.4% of clinical trials involving gene therapy are for cancer indications [23]. In solid tumours, in order to reach the cancer cells, gene therapeutics must go through a dense extracellular matrix formed mainly of collagen secreted by cancer-associated fibroblasts [24]. Moreover, the high interstitial fluid pressure (IFP) in solid tumours due to the leaky vasculature, the lack of lymphatic drainage and the rapid growth of cancer cells presents a barrier for the uptake of drugs, especially high molecular weight molecules such as gene therapeutics [25].

Furthermore, gene therapeutics must cross the complex molecular structure of the cellular membrane. Nucleic acids are high molecular weight molecules. Because of the phosphate group in their backbone, they are negatively charged and thus, highly soluble in water. These characteristics make them unable to cross biological membranes. The main mechanism of cellular internalization is endocytosis [26,27]. Gene therapeutics are internalized to intracellular vesicles called endosomes, which contain many digestive enzymes that can degrade nucleic acids. Moreover, endosomal pH acidifies progressively as they transition from early to late endosomes and finally to lysosomes, nucleic acids, especially DNA, are not stable at such a low pH [28]. Therefore, gene therapeutics must escape the endosomal pathway in its early stage to avoid being exposed to low pH and catalytic enzymes.

Finally, RNA-based gene therapeutics, such as silencing RNAs, find their site of action in the cytoplasm. On the contrary, DNA-based gene therapy strategies need to reach the nucleus, getting through a double nuclear membrane or a nuclear pore to find their target.

Consequently, the need for a delivery system that allows gene therapeutics to reach their site of action, avoid degradation, cross cellular membranes, escape or avoid the endosome and reach the nucleus is evident.

## 4. Gene Delivery Systems

**Figure 2:**
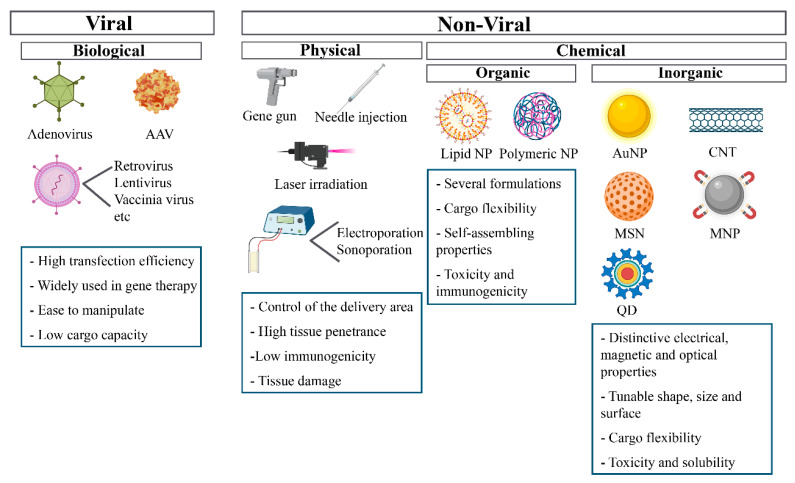
Representation of viral and non-viral delivery systems. AAV: adeno-associated virus. NP: nanoparticle. AuNP: gold nanoparticle. CNT: carbon nanotube. MNP: magnetic nanoparticle. MSN: mesoporous silica nanoparticle. QD: quantum dot.

Many delivery systems are being developed to accomplish a safe and effective delivery of gene therapy products (Figure 2). The potential of gene therapy as a treatment for many diseases has been known for a while, but the lack of optimal delivery systems has hindered its presence in the clinic.

### 4.1. Viral Vectors

An ideal viral vector is genetically stable, safe to handle, non-toxic for host cells, has a high packaging capacity and transduction efficiency, and does not elicit an immune response. Several viruses, such as retroviruses, lentivirus, adenovirus and adenovirus-associated viruses (AAV), have been widely studied to deliver gene therapeutics in diverse applications. Each of them has its unique characteristics, which affect their use as vehicles in gene therapy [29].

Although viral vectors have high transfection efficiency, several obstacles had been reported that compromise their use as delivery systems. It has been proved that adenovirus delivery systems can trigger host immunogenicity and cellular toxicity [30]. Integrating viral vectors, such as lentivirus and retrovirus vectors, fuse a fragment of their genetic material into the host cell genome, that increase the probability of insertional mutagenesis, which can lead to carcinogenesis, a drawback to adopt retroviruses and other integrating viral vectors as vectors for gene therapeutics [31,32,33].

Adenovirus-associated viruses (AVV) are viral systems that can infect a broad range of hosts, including dividing and non-dividing cells, without integrating with the host genome. Thus, the genetic material remains episomal. The main advantage is the low risk of genotoxicity, caused by insertional mutagenesis. Non-integrating viral vectors can provide stable transgene expression in non-dividing postmitotic cells, such as neurons, and transient or stable expression in dividing cells. If the stable expression in dividing cells is required, repeated dosing of non-integrating vectors may be an option, as long as the immune response can be managed. One limitation of AAV vectors is their small packaging size (~5.0 kb) compared with other viral vectors. However, several strategies have been investigated to enable the delivery of a large therapeutic gene [34].

### 4.2. Physical Methods

Non-viral gene delivery systems can be divided into physical and chemical techniques. Physical methods take advantage of physical phenomena that disrupt the cellular membrane and allow for the internalization of the genetic material. Physical methods include techniques, such as needle injection, in which naked DNA is directly injected through a syringe. This hydrodynamic injection is surprisingly effective for the in vivo transfection of genetic material into mice. [35]. Less invasive and new variants of this technique have been developed. These include the use of microneedles to deliver genetic material intradermally or in vivo intracellular injections for applications that only require the local uptake of small quantities of genetic material, such as vaccines [36,37].

Particle bombardment or gene gun is also a physical technique used to deliver gene therapeutics. In this method, gold nanoparticles are coated by DNA and propelled by pressurized helium gas against the target tissue. These accelerated particles can penetrate through cell membranes and several layers of cells in tissues [38,39].

Laser-assisted nucleic acid delivery is a technique in which laser energy creates microscopic holes in the cell membrane, enabling the genetic material’s internalization. An advantage of this technique is the ability to control the area of treatment and the energy delivered by modulating the laser wavelength and the duration of laser pulses [40,41].

Other techniques that use physical phenomena to facilitate gene delivery are electroporation and sonoporation, which induce pore formation and transient permeability using electric pulses or ultrasound, respectively [42,43]. Physical methods are attractive because of their low immunogenicity compared to viral vectors, but they can cause tissue damage.

### 4.3. Chemical Methods

#### 4.3.1. Organic Strategies

Chemical methods can be divided into organic and inorganic strategies. Organic strategies mainly include the use of cationic lipids or polymers that interact with negatively charged nucleic acids. Lipid-based drug delivery systems are one of the most attractive non-viral vectors for delivering of gene therapeutics as several formulations of these carriers have been approved by the FDA and EMA to deliver different drugs [44,45]. These systems take advantage of the self-assembling properties of amphiphilic lipids, such as phospholipids, to generate carriers that protect nucleic acids. Lipoplexes are electrostatic complexes spontaneously formed when liposomes composed of cationic lipids such as DOTAP (dioleoyl-3-trimethylammonium propane) interact with negatively charged oligonucleotides [46,47]. However, liposomes are highly dynamic systems that lack stability, which can significantly impact nucleic acid encapsulation, causing the genetic material’s release before its arrival to the site of action.

After the recent success of SARS-CoV-2 vaccines, lipid nanoparticles have become widely known vectors for delivering genetic material [48,49]. These spherical vesicles are composed of ionizable lipids that, due to their positive charge at low pH, allow the interaction with nucleic acids through electrostatic forces and the endosomal escape once they are internalized by cells. In addition, these lipids are neutral at physiological pH, which reduces their toxicity and immunogenicity. As a result, lipid nanoparticles have allowed for the delivery of the first RNAi therapeutic approved by the FDA [50]. Additionally, it has been demonstrated that lipid nanoparticle systems can deliver CRISPR/Cas9 components to achieve clinically relevant levels of genome editing in vivo [51].

Exosomes are extracellular vesicles naturally secreted by numerous cells with a size range of 40 to 160 nm in diameter. These vesicles mediate intercellular communication by interchanging many different cell components, including DNA, RNA, proteins and other metabolites between cells. Their natural biocompatibility and minimal immune clearance have driven extensive research to exploit these carriers as gene delivery vectors [52,53]. Exosomes differ from liposomes in that exosomes have a more complex composition. In addition, their lipid membrane is rich in proteins that allow for more specific targeting and provides higher stability [54]. However, the use of these systems as gene delivery vectors is hindered by difficulties in production, isolation and purification.

Polymer-based systems have also been widely studied for the delivery of gene therapeutics. Positively charged polymers, such as poly-ethylenimine (PEI) or chitosan, can form nanoparticles, called polyplexes, upon interaction with negatively charged nucleic acids [55]. In addition, novel polymerizable pH-sensitive surfactants are being developed. These carriers can form stable nanoparticles with nucleic acids and disrupt membranes in a pH-sensitive manner, enabling endosomal escape [56]. Lipoplexes and polyplexes can achieve high in vitro transfection. However, their use in vivo is hindered by their toxicity and immunogenicity [57,58,59,60,61].

Most of these systems have a positively charged surface leading to interaction with negatively charged membranes and proteins in physiological media. The positively charged surface consequently prompts the recognition and uptake of these systems by phagocytic cells and results in activation of the immune response [62].

Novel gene delivery systems are being developed within the cutting-edge field of DNA and RNA nanotechnology. Nucleic acid molecules can now be produced and manipulated easily and have great structural flexibility. The specific sequences of these molecules can be designed to predictably fold by complementarity into specific shapes and self-assemble into supramolecular modular structures [63,64]. DNA and RNA nanostructures are naturally biocompatible and negatively charged, which minimizes toxicity and off-target effects. Chemical modifications that have been introduced in the nucleic acid backbone confer resistance to degradation by nucleases [65].

#### 4.3.2. Inorganic Strategies

Inorganic nanomaterials are an emerging tool for therapeutic delivery because of their different structural and physical properties. Nanostructures’ size, shape and surface can be tailored depending on their needs, making them a helpful approach in almost every gene delivery case [66]. Inorganic nanoparticles present unique optical, physical, electrical and magnetic properties, depending on the base material. Gold nanoparticles, magnetic nanoparticles, carbon nanotubes, quantum dots and silica nanoparticles are the most commonly used for gene delivery or as a therapeutic tool [67,68,69].

##### Gold Nanoparticles (AuNPs)

AuNPs exhibit photothermal properties, which are used as a biomedical application in different diseases such as cancer [70]. There are several types of AuNPs depending on the shape, such as nano-spheres, nano-rods, nano-stars, nano-shells and nano-cages. These nanoparticles are the most studied because of their (I) high biocompatibility, (II) ease of surface modification, (III) ability to conjugate with biological ligands, such as polymers or genetic material, (IV) ease of molecular imaging. The AuNP surface can be treated to improve its stability and specificity and to reduce aggregation. Functionalized AuNPs are used for gene transfection and silencing, targeted drug or gene delivery, intracellular detection, bioimaging, cancer studies, and as biosensors [71,72,73,74]. Unveiling the applications of AuNPs depending on the size and shape to design the most accurate delivery system is still a challenge that needs to be further explored.

##### Magnetic Nanoparticles (MNPs)

MNPs are synthetically produced using mainly two approaches: (I) breaking down materials to the nanoscale size and (II) forming MNPs from the nucleation of atoms and growth process. In addition, MNPs can be functionalized with different moieties to target specific cells or molecules. Thus, there are two main mechanisms of delivery: (I) passive targeting that occur via enhance permeability and retention and (II) active targeting, which targets the desired localization by functionalizing the MNP or using a magnetic field. (see “What happens when nano enters the body?”).

Usually, in biomedicine, the core of the MNPs is made by magnetite (Fe3O4) or maghemite (γ-Fe2O3) which is synthesized chemically, and finally, the base material is coated to ensure the colloidal stability and the specificity of the MNP. The properties of the MNPs strongly depend on the shape and the size of the nanoparticles. MNPs are a promising therapeutic tool that could be used in several medical approaches, such as (I) targeted drug delivery in cancer theranostic, (II) MRI-assisted drug delivery, (III) magnetically guided drug delivery, and (IV) stimuli-responsive drug delivery, although more research is needed to optimize the delivery methodology in combination with the detection modality [75,76,77,78].

##### Carbon Nanotubes (CNT)

CNTs are rolled-up sheets of carbon atoms that form single or multi walled cylinders. They exhibit exceptional electrical, thermal, mechanical and optical properties. CNTs are highly hydrophobic, and the shape tends to form bundles altogether, contributing to cell toxicity. However, functionalizing the CNT surface seemed to reduce its toxicity either via covalent or non-covalent modifications.

One of the multiple advantages of using CNTs is the large loading capacity compared to spherical shape nanomaterials. The ease functionalization allows CNTs to be targeted to specific cells or molecules. Moreover, they allow for multiple functionalization which is a useful property in terms of targeting and delivery. Another characteristic of CNTs is their ability to absorb light at an infrared wavelength which could be useful for tracking the delivery and for imaging. CNTs offer high loading capacity, improved biocompatibility, targeting and tracking ability. This combination of properties makes them an excellent nanomaterial for gene delivery with great potential in nanomedicine [79,80].

##### Quantum Dots (QDs)

Conventional QDs are semiconductor crystals with unique optical and electronic properties that depend on their size and shape. Since different types of QDs can be excited with the same wavelength and emit light at different wavelengths depending on the material, shape, and size. This property has led to the development of many QDs formulations [81,82].

The multiple characteristics such as high brightness, resistance to photobleaching, multiplexing capacity, and high surface-to-volume ratio make them excellent candidates for intracellular tracking, diagnostics, in vivo imaging, and therapeutic delivery. QDs have been used to facilitate gene therapy through intracellular delivery and imaging of treatment with small interfering RNA (siRNA). On the other hand, QDs show several limitations: low aqueous solubility, complicated surface chemistry, low biological specificity, poorly controlled biodistribution to target tissues, and the potential for severe long-term toxicity [82,83].

A new group of QDs, called natural carbon-based quantum dots (NQDs), has recently emerged in the biomedical field. These novel QDs are natural carbon QDs that exhibit several advantages: abundance, eco-friendly nature, aqueous solubility, photo-stability, diverse functionality, and biocompatibility in comparison with other conventional quantum dots (CQDs). Based on their advantages, multiple NQD applications, focused on drug delivery, have been developed. These include their use as sensing and tracing probes, photo-activated antimicrobial agents, anticancer drug delivery systems, antioxidants, and sensors for neurodegenerative diseases [84].

##### Mesoporous Silica Nanoparticles (MSNs)

MSNs have large pore volume and area, tuneable pore size and shape, robustness, and facile surface functionalization. These advantages make MSNs an amenable nano-system to carry small and large molecules such as proteins or even DNA. Although MSNs are hardly degraded in the body, it seems that MSNs exhibit low toxicity and are biocompatible. Nevertheless, variations of their shape, size, surface chemistry, administration dose and functionalization, can have a great impact on their toxicity. MSNs are promising delivery systems that have been used for (I) improvement of drug solubility, (II) selective targeting for localized therapy, (III) controlled dosage and smart behaviour (internal and external stimuli-responsive drug delivery) and (IV) theranostics [85,86]. (See “Future perspectives on nanotechnology-based gene delivery”.)

In the field of inorganic nanoparticles, there are other emerging methodologies, such as nanodiamonds. Because of their inexpensive, large-scale synthesis, the potential for surface functionalization, and high biocompatibility, nanodiamonds are being investigated as a potential material in biological applications [87]. Improving the current delivery systems to avoid toxicity and enhance the delivery process and finding new inorganic nanoparticles suitable for gene delivery is still a challenge.

## 5. What Happens When Nano Enters the Body?

### 5.1. Protein Corona

**Figure 3:**
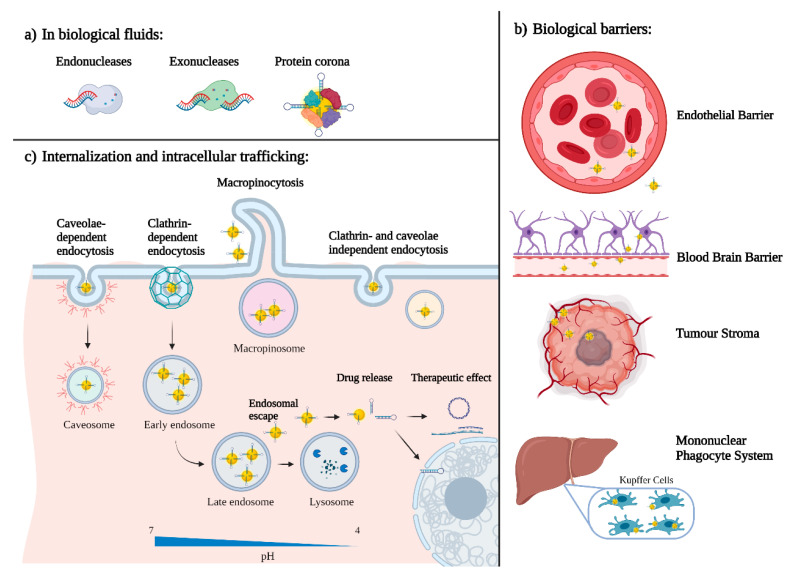
Biological barriers for nanotechnology-based gene delivery systems: (**a**) at the organ/tissue level, (**b**) at the molecular level (**c**) and at the cellular level.

When nanotechnology-based delivery systems reach systemic circulation, they interact with the biomolecules present in blood, mainly with proteins that bind to the nanoparticle surface and form the so-called protein corona (Figure 3a) [88]. Protein absorption is governed by van der Waals, hydrophobic and electrostatic forces. The composition of this protein corona is highly dynamic and dependent on the charge, size and hydrophobicity of the nanoparticle surface [89].

Depending on the composition, the protein corona can have different effects on the fate of nanoparticles. Some proteins, such as complement factors and immunoglobulins, called opsonins, can facilitate the uptake of nanoparticles by the phagocytic system leading to a decreased half-life and the activation of the immune response [90,91]. Opsonisation induces nanoparticle uptake by the mononuclear phagocyte system, including Kupffer cells and macrophages in the liver and spleen. Opsonisation leads to an evident biodistribution of nanoparticles that accumulate in these organs.

On the contrary, protein corona formation can have certain advantages if formed by specific proteins. It can help the nanoparticle evade the immune system because, if non-immunogenic self-proteins envelop the nanoparticle, it will hide the nanoparticle’s surface and not trigger an immune response. The protein corona, in some cases, can also increase the uptake of the nanoparticles by target cells. For example, if the nanoparticle is coated with albumin, this can increase internalization into cancer cells. Tumours naturally uptake higher quantities of albumin than normal cells because they need nutrients to sustain their rapid growth [92]. Furthermore, it is possible to modulate the surfaces of nanoparticles to increase interaction with selected proteins such as albumin [93].

Nanoparticles with a hydrophobic or positively charged surface tend to be opsonized more efficiently in the bloodstream. Thus, it is desirable to avoid forming the protein corona to provide stealthiness to the nanoparticles and avoid recognition and uptake by macrophages in most cases. The primary strategy to achieve stealthiness is by grafting polyethylene glycol (PEG) to the surface of nanoparticles. PEG is a hydrophilic polymer that sterically reduces the interaction of proteins with the nanoparticle surface, increases circulation time by preventing macrophage uptake and increases nanoparticle stability, avoiding aggregation. The PEGylation of proteins and oligonucleotides has been widely used, and it has been approved by the Food and Drug Administration (FDA) [94]. However, it has been recently reported that PEG can be immunogenic, and individuals treated with PEGylated compounds can produce antibodies against PEG. The presence of anti-PEG antibodies can increase drug clearance and cause hypersensitivity and severe side effects [95]. Therefore, several alternatives to PEG are being developed, such as polysarcosine, a biodegradable, more biocompatible, less immunogenic polypeptoid [96].

### 5.2. Active and Passive Targeting

Furthermore, the protein corona is not the only reason that nanoparticles tend to accumulate mainly in the liver, but also their natural size. The liver and spleen have a discontinuous endothelium that allows for the extravasation of nanoparticles without having to cross this barrier. This fenestrated vasculature is also characteristic of most solid tumours. The process of creating new blood vessels, called angiogenesis, which happens in most solid tumours, is far from perfect and leads to the presence of similar gaps in the endothelium. Nanoparticles are thus able to easily extravasate and enter the tumour. Moreover, the lack of lymphatic drainage facilitates nanoparticle accumulation. This phenomenon is called the enhanced permeation and retention (EPR) effect and was discovered by Maeda in 1989 [97]. Even though the EPR has been widely used for passive targeting of nanoparticles, its effectiveness has recently aroused substantial controversy. Recently, clinical and preclinical studies have established the heterogeneity of this phenomenon [98]. The EPR effect in tumours differs depending on their tissue of origin, size and vascular density. The high tumour interstitial fluid pressure and complex extracellular stroma (Figure 3b), characteristic of most solid tumours, can negatively impact the passive targeting of nanoparticles. Recently, it has been demonstrated that an active delivery mechanism based on cellular transcytosis may be more dominant than passive EPR-based nanoparticle accumulation, thus creating a strong need for in vivo nanoparticle delivery and tumour penetration. These results challenge our current methods for developing cancer nanomedicine and suggest that understanding these active pathways will unlock strategies to enhance tumour accumulation [99,100].

To selectively deliver gene therapeutics to organs other than the liver, spleen or solid tumours, it is essential to consider alternative targeting strategies. A widely studied approach to increase nanoparticle uptake by targeted cells is active targeting. It consists of grafting targeting moieties to the nanoparticle surface, such as antibodies, aptamers, peptides, sugars or other biomolecules that are recognized by receptors differentially expressed on the surface of the targeted cells [101]. Nanobodies are a new type of targeting moieties being developed; these are single-domain antibodies that are more stable and versatile than traditional antibodies and display much lower intrinsic immunogenicity [102].

### 5.3. Internalization and Intracellular Trafficking

The interaction of targeting moieties with receptors on the cellular membrane usually triggers nanoparticle internalization via endocytosis. Endocytosis is a cellular process by which invaginations of the cellular membrane are formed, engulfing extracellular substances that then become vesicles that enter the cell’s cytoplasm (Figure 3c). There are different pathways by which endocytosis occurs. One of them is micropinocytosis, a non-selective uptake in which a large volume of extracellular fluid is internalized in large endocytic vesicles, called macropinosomes. Another endocytosis mechanism is clathrin-mediated endocytosis; this is a selective process in which vesicles of an average size of 120 nm are formed and follow the endo/lysosomal route. Thus, particles internalized by this mechanism require an endosomal escape strategy. Furthermore, endocytosis can be caveolae-dependent, which is also a selective mechanism. In this case, invaginations have an average size of 50–60 nm and, when internalized, form a caveosome, which bypasses the endo/lysosomal pathway, and are delivered to the endoplasmic reticulum and nucleus. Finally, endocytosis can be clathrin- and caveolae-independent, a poorly understood pathway [103].

As previously mentioned, nanoparticles internalized by clathrin-mediated endocytosis are directed to intracellular vesicles, known as endosomes. The pH in these vesicles acidifies as they transition from early to late endosomes and finally to lysosomes. For example, early endosomes have a pH of 6–6.5, whereas in lysosomes the pH ranges from 5 to 4.5. At this pH, hydrolases are activated and degrade proteins and nucleic acids.

In order to avoid the degradation of gene therapeutics and allow them to reach their intracellular target, several strategies to escape endosomes in the early stage are being developed. These include the use of fusogenic lipids in liposome formulations [104] or encapsulating nucleic acids in pH-sensitive lipoplexes or polyplexes, which are able to disrupt biological membranes when the pH acidifies [105,106,107]. Other strategies include the functionalization of nanoparticles with cell-penetrating peptides (CPPs), short peptides that facilitate cellular uptake, such as the TAT peptide discovered from the HIV-1 Tat protein [108]. CPPs usually contain either positively charged amino acids, polycationic peptides, alternating polar and non-polar amino acids, amphipathic peptides or non-polar amino acids, hydrophobic peptides [109]. In addition, pH-sensitive molecules that are protonated at acidic pH have also been used to facilitate the endosomal escape of nanoparticles. These molecules become amphiphilic in acidic pH and interact with membrane phospholipids and introduce high buffering capacity that causes membrane disruption by the proton-sponge effect [56]. However, these mechanisms are non-selective, and they often present toxicity issues. Thus, there is a need to develop nanoparticles that allow alternative internalization mechanisms.

### 5.4. Nanoparticles Pharmacokinetics and Clearance

Even though several strategies are being studied to develop biodegradable inorganic nanoparticles, most of the materials used nowadays do not degrade in the body. Therefore, it is essential to understand the clearance pathways to avoid bioaccumulation [110,111].

Nanoparticles physicochemical properties can influence their clearance from the circulation, but it is highly dependent on the interactions with the MPS or reticuloendothelial system [71]. Cationic nanoparticles are generally most rapidly cleared in terms of surface charge, followed by anionic nanoparticles, whereas neutral and slightly negative nanoparticles have the most prolonged half-lives in circulation. Some nanoparticles designs implement surface modifications such as peptides, PEG, or cell membrane coatings to reduce these interactions with phagocytic cells [18]. Relating their size, for example, nanoparticles with a diameter less than 10 nm have generally been shown to be rapidly eliminated by the kidneys, whereas nanoparticles larger than 200 nm risk activating the complement system [112,113].

For inorganic nanoparticles, size, shape, charge, and surface functionalization can be modified, enabling tailored designs to obtain more desirable pharmacokinetic profiles [114,115].

## 6. Future Perspectives on Nanotechnology-Based Gene Delivery

Nanotechnology-based gene delivery for gene therapy represents a significant step towards personalized medicine, creating a change in our ability to treat and potentially even cure many intractable illnesses. Each gene therapy is designed based on detailed information about the roots of a patient’s disease. Since gene therapy emerged as a potential approach for personalized medicine, the urge to develop a system with the capacity of cell/tissue selective targeting and delivery of therapeutic genetic material has become of significant priority [116,117].

**Figure 4:**
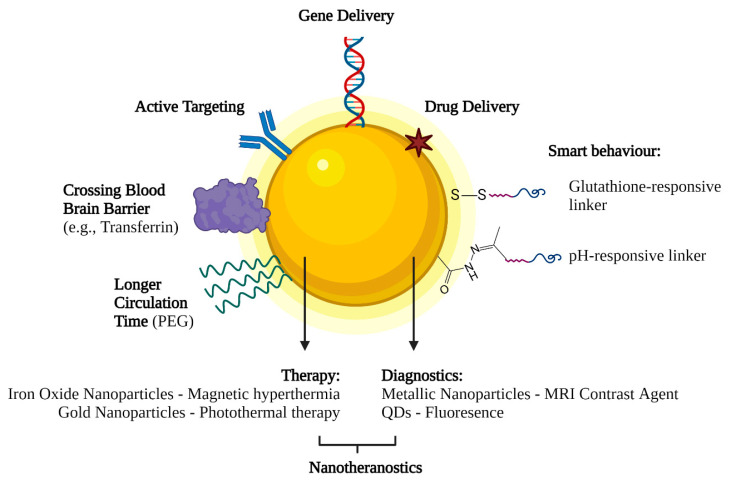
Multifunctionalities of nanotechnology-based delivery systems.

Nowadays, nanotechnology-based delivery systems are starting to be commonly used for gene therapy. One of the main advantages is their incredible versatility. Because of their high surface to volume ratio, nanoparticle surfaces can be functionalized with multiple moieties that allow them to be carriers of gene therapeutics and encompass other capabilities (Figure 4). For example, grafting specific ligands to the nanoparticle’s surface, such as transferrin, may allow them to cross the blood–brain barrier and deliver gene therapeutics to the central nervous system, which currently presents a significant challenge. This versatility allows nanoparticles to be used for various applications, such as in cancer treatment [118].

One of the most critical applications for nano-base gene delivery is the use of nanoparticles in genetic-based vaccines [119,120]. The use of particles as nanoplatforms displaying relevant antigenic moieties is appealing as an alternative approach to conventional vaccines. Many particles are currently evaluated as antigen carriers, including inorganic and polymeric nanoparticles, virus-like particles (VLPs), liposomes, and self-assembled protein nanoparticles. The advantages of these materials reside primarily in their size. Incorporation of antigens in nanoparticles can be achieved by encapsulation (physical entrapment) or by conjugation (covalent functionalization) [121]. Studies have demonstrated that nanoparticles could protect the native structure of antigens from proteolytic degradation and improve antigen delivery to antigen-presenting cells (APCs) [122].

Nanoparticles can be administered via subcutaneous and intramuscular injections or can be delivered through the mucosal sites (oral and intranasal) and penetrate capillaries as well as mucosal surfaces [123].

In addition, nanoparticles incorporating antigens can exert a slow local release of antigens when attracting antigen-presenting cells relative to a fast release, ensuring prolonged antigen presentation to immune cells. A new framework for vaccine adjuvant development [124].

For instance, nanoparticles such as carbon nanotubes (CNTs), carbon black nanoparticles, poly (lactic-co-glycolic acid) (PLGA) and polystyrene nanoparticles, titanium dioxide (TiO2) nanoparticles, silicon dioxide (SiO2) nanoparticles, and aluminium oxyhydroxide nanoparticles have been reported to induce NLRP3-associated inflammasome activation [125]. Overall, nanoparticles are promising antigen carriers and immune cell activators for vaccination.

Like in the case of vaccine and cancer delivery systems, one of the most relevant advantages of nanotechnology-based systems is that they can deliver their cargo in response to specific internal or external stimuli. This behaviour is called “smart”, and it refers to the ability of these systems to control the release of the therapeutic agents in space and time [126]. These stimuli can be physical, such as temperature, electric and magnetic fields or light. For instance, superparamagnetic nanoparticles can increase their temperature under an alternating magnetic field. This controlled temperature increase can trigger localized hyperthermia and the release of gene therapeutics [127]. In addition, AuNPs have been engineered to serve as targeted delivery vehicles, molecular probes and sensors. Their small size and surface characteristics enable them to access the tumour microenvironment (TME). Moreover, the stimuli-responsive properties (response to hypoxia and acidic pH) of nanoparticles to TME enable the development of AuNPs as potential therapeutic and diagnostic tools [128].

Additionally, these stimuli can have a chemical nature, including pH and redox state, which can trigger the release of gene therapeutics. For example, this strategy can be used when targeting tumours. The extracellular tumour microenvironment has a slightly more acidic pH than normal tissue due to its unique cellular metabolism that favours fermentation. Thus, nanoparticles tuned to release genetic material at this pH can target tumours selectively [129].

Different biomolecules and enzymes can also facilitate the release of gene therapeutics from their carrier. For example, glutathione, which is 100 to 1,000 times higher in intracellular than extracellular environments, can be used to release the genetic material [130]. In addition, enzymes specifically present in the targeted tissue or cell types can also be used to trigger gene release at specific sites [131].

Furthermore, nanoparticles allow for combination therapy, a treatment modality that combines two or more therapeutic agents. The basis of, for instance, a more efficient cancer therapy [132]. Moreover, these multifunctionality characteristic of nano-systems allow the combination of therapy with diagnostics. This strategy is called theranostics and can be achieved mainly with inorganic nanoparticles. For example, super-paramagnetic iron oxide nanoparticles and magnetic mesoporous silica nanoparticles can be used as contrast agents for MRI and can deliver gene therapeutics, making them great theranostic agents for solid tumours [133,134,135,136].

## 7. Conclusions

Although there is no doubt that novel nanotechnological methods for gene delivery will rapidly improve our current capability, some limitations are still to be resolved for their use as gene delivery systems, such as potential toxicity and low transfection efficiency. Undeniably, the further understanding of different cell metabolism, cell structure and microenvironment, the development of novel nanomaterials with specific properties at the nanoscale will be essential to overcome these limitations.
